# Benefits of lymphadenectomy for upper tract urothelial carcinoma only located in the lower ureter: a bicentre retrospective cohort study

**DOI:** 10.3389/fonc.2023.1115830

**Published:** 2023-04-14

**Authors:** Yupeng Cui, Youyi Lu, Jitao Wu, Changyi Quan

**Affiliations:** ^1^ Department of Urology, The Second Hospital of Tianjin Medical University, Tianjin, China; ^2^ Department of Urology, The Affiliated Yantai Yuhuangding Hospital of Qingdao University, Yantai, Shandong, China

**Keywords:** upper tract urothelial carcinoma, lymphadenectomy, nephroureterectomy, recurrence-free survival, cancer-specific survival

## Abstract

**Background:**

Upper tract urothelial carcinoma (UTUC) is a rare and highly malignant urothelial tumor originating from the renal pelvis and ureter associated with poor prognosis. It has been established that 70% of ureteral tumors occur in the lower ureter. Radical nephroureterectomy (RNU) with ipsilateral bladder cuff excision is regarded as the standard treatment for UTUC. Current evidence supports the role of lymph node dissection (LND) in determining tumor staging, but no consensus has been reached on the potential survival benefits. The present study retrospectively analyzed cases of UTUC limited to the lower ureter to evaluate the survival benefits of LND during RNU.

**Methods:**

The present study retrospectively analyzed data from patients with UTUC limited to the lower ureter from two medical centers from 2000 to 2016 and assessed the survival outcomes, including recurrence-free survival (RFS) and cancer specific survival (CSS). During subgroup analysis, we stratified by pathological tumor (pT) stages and postoperative adjuvant chemotherapy (AC).

**Results:**

The study cohort included 297 patients separated into LND (n=111) and non-LND (n=186) groups. The two groups were comparable except for the pathological N stage. The LND group was associated with superior survival in terms of RFS (27.0% vs. 18.3%, p=0.044) and CSS (53.2 vs. 39.8%, p=0.031) compared to the non-LND group (n=186). In pT2-4 patients, the LND group was associated with better 3-year RFS (50.5% vs. 32.3%, p<0.05), 5-year RFS (29.7% vs. 12.0%, p<0.05), and overall RFS (18.7% vs. 6.0%, p<0.05) than the non-LND group. Besides, the LND group was associated with a significantly better 3-year CSS (68.1% vs. 49.6%, p=0.003), 5-year CSS (51.6% vs. 30.8%, p<0.05) and overall CSS (45.1% vs. 24.1%, p<0.05). In patients that underwent AC, the LND group had better survival benefits in terms of RFS (29.4 vs. 16.7%, p=0.023) and CSS (52.9% vs. 40.5%, p=0.038) compared to the non-LND group.

**Conclusion:**

LND has survival benefits in patients with UTUC localized to the lower ureter, especially for≥pT_2_ stage UTUC and AC cohorts. Overall, the therapeutic effect of LND in UTUC cannot be replaced by AC.

## 1 Introduction

Urothelial carcinoma (UC) is the fourth most common male tumor and may involve different parts of the urinary system, including the renal pelvis, ureter, bladder or urethra. Upper tract urothelial carcinoma (UTUC) is a rare and highly malignant tumor originating from the renal pelvis and ureter, accounting for 5-10% of all UC cases (1). Due to the absence of clinical symptoms during the early stages and the thin muscular layer and secluded location of the upper urinary tract, UTUC is prone to invasive growth. About two-thirds of patients have advanced-stage disease upon diagnosis, resulting in poor treatment efficacy and prognosis (2).

According to the initial location, UTUC can be subdivided into four segments: renal pelvis, upper, middle and lower ureter. Current evidence suggests that the incidence of UTUC has gradually increased over the past decades, with a significant decrease in renal pelvic cancers (1.19 to 1.15) and an increase in ureteral disease (0.69 to 0.91) (3). About 70% of ureteral tumors occur in the lower ureter, 25% in the middle ureter, and 5% in the upper ureter (4). An increasing body of evidence suggests that lower ureteral tumors have the worst prognosis (5–7).

Radical nephroureterectomy (RNU) with ipsilateral bladder cuff excision is regarded as the standard treatment for UTUC (2). However, it has been reported that the prognosis for UTUC patients remains poor after RNU surgery, and the five-year survival rate is very low (8). Current evidence supports the role of lymph node dissection (LND) in determining tumor staging, but no consensus has been reached on the potential survival benefit (9). Studies from different centers have yielded inconsistent results (9–11). Accordingly, no guidelines recommend routine LND during RNU in this patient population.

Herein, we retrospectively analyzed cases of UTUC localized to the lower ureter to evaluate the survival benefits of LND during RNU.

## 2 Methods

### 2.1 Patients

In this retrospective cohort study, we initially identified 1379 patients with UTUC that underwent open or laparoscopic RNU with/without LND at the Yantai Yuhuangding Hospital Affiliated to Qingdao University (n=917) and the Second Hospital of Tianjin Medical University (n=462) from January 2000 to January 2016.

**Figure 1:**
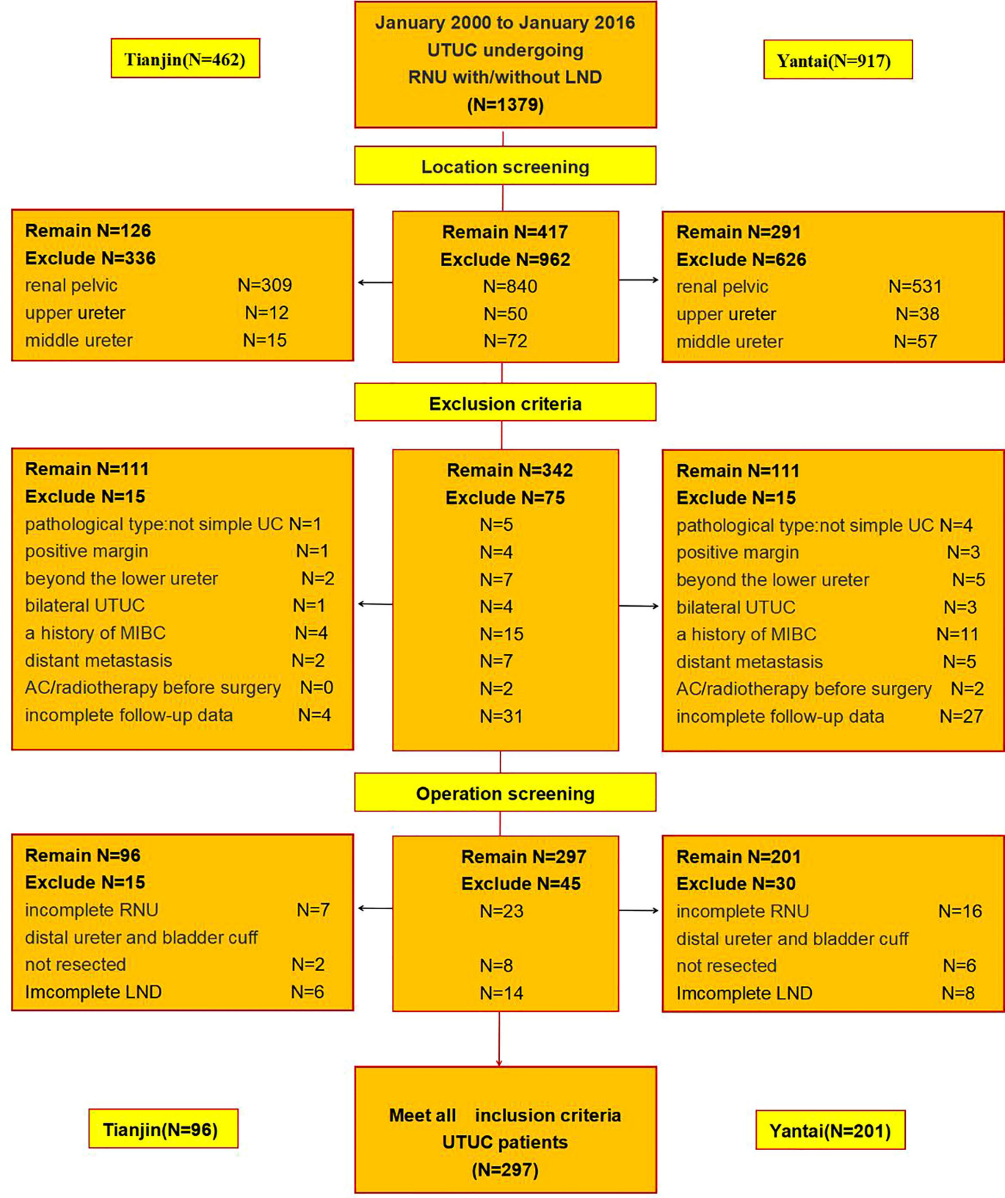
Flow diagram of the selection process.

417 patients with tumors of the lower ureter were obtained after excluding 962 patients with tumors involving the renal pelvis (n=840) and upper (n=50) and middle (n=72) ureters. 75 ineligible patients were excluded based on the exclusion criteria, including 1. the pathological type is not simple urothelial carcinoma (n=5); 2. positive margins (n=4); 3. the boundary of the tumor beyond the boundary of the lower ureter(n=7); 4. bilateral ureteral tumors (n=4); 5. history of muscle-invasive bladder cancer (MIBC) (n=15); 6. distant metastasis before operation(n=7); 7. adjuvant chemotherapy or radiotherapy before surgery (n=2); 8. missing or incomplete follow-up data (n=31). Finally, we excluded cases with incomplete RNU (n=23), incomplete LND (n=14), and distal ureter and bladder cuff not resected by an open incision (n=8) (**Figure 1**). Ultimately, 297 patients comprised the final study population.

All patients underwent computed tomography urography (CTU) and CT angiography (CTA) or magnetic resonance hydrography of the urinary system (MRU), cystoscopy or diagnostic ureteroscopy, and urinary cytology before surgery.

The present study was conducted upon approval from the Institutional Review Board of both medical centers.

### 2.2 Surgical procedures

All patients underwent standard RNU with ipsilateral bladder cuff resection. RNU surgeries were performed through laparoscopic or open approach by two experienced surgical teams in each center. The distal ureter and the bladder cuff were treated by an open incision in the lower abdomen. LND was performed through this incision. LND involved ipsilateral pelvic lymph nodes, including lower 1/3 of periureter lymph tissues, common iliac lymph nodes, internal iliac lymph nodes, external iliac lymph nodes, and obturator lymph nodes.

All surgical specimens were processed according to standard pathological procedures. Two experienced anatomical pathologists reviewed all slides. Any disagreements were resolved by consensus-based discussion or by a third pathologist. All tumors were graded according to the 2004 World Health Organization/International Society of Urological Pathology (WHO/ISUP) consensus classification and staged according to TNM classification 2017 for UTUC. N_0_ was defined when the scope of dissection was sufficient, the number of lymph nodes was not less than 8, and all nodes were negative. Otherwise, it was excluded and regarded as incomplete LND.

Patients in both groups were routinely given intravesical instillation of 30mg piroxacin 1 week after surgery, once a week for 8 weeks, and then once a month for 6-12 months in total, to prevent tumor recurrence. Transurethral resection of bladder tumor was performed for non-muscle-invasive bladder cancer (NMIBC) postoperative recurrence.

### 2.3 Adjuvant treatment

None of the patients received radiation therapy after surgery. Adjuvant chemotherapy (AC) was performed mainly due to locally advanced disease (pT_2-4_/N_0-2_/M_0_ or pT_any_/N_1-2_/M_0_) after the surgeon and patients reached a consensus. The adjuvant chemotherapy scheme was based on cisplatin.

### 2.4 Follow-up

During follow-up, patients generally underwent cystoscopy and urinary cytology every three months for the first two years, chest and abdomen CT every 6 months and annually thereafter. A bone scan was performed when bone metastasis was suspected clinically. 31 patients were lost to follow-up at the end of the study since they could not be contacted or declined to follow-up.

### 2.5 Statistical analysis

The clinical and pathological characteristics of the two groups were compared using Student’s t-test for continuous variables and the chi-square test for categorical variables. Survival probabilities were estimated using the Kaplan-Meier method, and the log-rank test was applied to compare survival curves. Univariate and multivariate Cox proportional hazards regression analyses were conducted to evaluate the association between prognostic factors and survival outcomes. All statistical analyses were performed using SPSS version 21.0, and P values <0.05 were statistically significant. The primary end-points were recurrence-free survival (RFS) and cancer-specific survival (CSS). Local recurrence included cancer recurrence within the resection area regional lymph nodes, or peritoneal recurrence. Concomitant regional and distant metastases were treated as distant recurrences. RFS was measured from the date of surgery until recurrence.

## 3 Results

### 3.1 Characteristics of patients

The final study cohort comprised 297 patients who underwent standard RNU and ipsilateral bladder cuff resection with (n=111, 37.4%) or without LND (n=186, 62.6%).The median follow-up time in the LND and non-LND groups was 47.8 ± 26.6 (range 4-91) and 40.2 ± 27.1 (range 4-92) months, respectively. The clinicopathological characteristics of the study cohort (LND vs. non-LND) are presented in **Table 1**.

**Table 1:** Baseline characteristics of the study cohort and comparative analysis results according to surgical method.

Clinical Parameters	Total (n=297)	non LND (n=186)	LND (n=111)	P value
**Age, Median (Range),year**	63.61 (30-81)	63.86 (31-81)	63.20 (30-81)	0.60^a^
**Gender, n (%)**				0.92^b^
Male	199 (67.0)	125 (67.2)	74 (66.7)	
Female	98 (33.0)	61 (32.8)	37 (33.3)	
**Laterality**				0.75^b^
Left	156 (52.5)	99 (53.2)	57 (51.4)	
Right	141 (47.5)	87 (46.8)	54 (48.6)	
**Hydronephrosis,n (%)**				0.66^b^
Yes	169 (56.9)	104 (55.9)	65 (58.6)	
No	128 (43.1)	82 (44.1)	46 (41.4)	
**Urinary Cytology,n (%)**				0.40^b^
Positive	127 (42.8)	83 (44.6)	44 (39.6)	
Negative	170 (57.2)	103 (55.4)	67 (60.4)	
**NMBIC,n (%)**				0.92^b^
Yes	82 (27.6)	51 (27.4)	31 (27.9)	
No	215 (72.4)	135 (72.6)	80 (72.1)	
**Tumor Size, Mean (SD),cm**	2.81 (0.91)	2.78 (0.89)	2.87 (0.96)	0.39^b^
**Tumor Multifocality, n (%)**				0.85^b^
Unifocal	250 (84.2)	156 (83.9)	94 (84.7)	
Multifocal	47 (15.8)	30 (16.1)	17 (15.3)	
**LVI,n (%)**				0.27^b^
Yes	151 (50.8)	90 (48.4)	61 (55.0)	
No	146 (49.2)	96 (51.6)	50 (45.0)	
**Tumor grade, n (%)**				0.78^b^
Low	104 (35.0)	64 (34.4)	40 (36.0)	
High	193 (65.0)	122 (65.6)	71 (64.0)	
**pT Stage, n (%)**				0.32^b^
PTa/Tis	22 (7.4)	16 (8.6)	6 (5.4)	
pT1	51 (17.2)	37 (19.9)	14 (12.6)	
pT2	114 (38.4)	68 (36.5)	46 (41.5)	
pT3	87 (29.3)	53 (28.5)	34 (30.6)	
pT4	23 (7.7)	12 (6.5)	11 (9.9)	
**pN Stage, n (%)**				–
pN0	51 (17.2)	–	51 (45.9)	
pNx	186 (62.6)	186 (100.0)	–	
pN+	60 (20.2)	–	60 (54.1)	
**No. of dissected LN in LND, mean (SD)**			10.57 (1.18)	–
**No. of positive LN in LND, mean (SD)**			2.31 (2.66)	–
**LN+ density in LND, mean (SD)** **(positive/total lymph nodes)**			21.87 (25.41)	–
**Size of positive LN in LND, n (%)**				–
≤2cm			166 (64.8)	
>2cm			90 (35.2)	
**Surgical Approach, n (%)**				0.95^b^
Laparoscopic RNU	236 (79.5)	148 (79.6)	88 (79.3)	
Open RNU	61 (20.5)	38 (20.4)	23 (20.7)	
**Adjuvant Chemotherapy, n (%)**				0.06^b^
Yes	78 (26.3)	42 (22.6)	36 (32.4)	
No	219 (73.7)	144 (77.4)	75 (67.6)	
**Hospital, n (%)**				0.82^b^
YanTai	201 (67.7)	125 (67.2)	76 (68.5)	
Tianjin	96 (32.3)	61 (32.8)	35 (31.5)	

LND, lymph node dissection; NMIBC, non muscle-invasive bladder cancer; LVI, lymphovascular invasion; LN, lymph node; Tis, carcinoma in situ; RNU, radical nephroureterectomy; SD, standard deviation. Bold values indicate that P-value < 0.05, and considered statistically significant. ^a^student’s test ^b^chi-square test.

Overall, the median age of the patients was 63.61 (30-81years). The male-to-female ratio was approximately 2:1. Nearly three-quarters of the patients (LND 82%, non-LND 71.5%) presented with invasive disease (≥pT_2_ stage), and two-thirds had high-grade features. Lymphovascular invasion (LVI) was found in about half of the patients. Of the 297 eligible patients, 201 cases were from Yantai, and 96 from Tianjin. 236 (79.5%) patients underwent laparoscopic RNU, and 61 (20.5%) patients underwent open RNU. Approximately a quarter of the patients (LND 32.4%, non-LND 22.6%) were treated with postoperative adjuvant chemotherapy. No significant differences were observed between the two groups except for the pathological N stage.

### 3.2 Survival outcomes in the entire cohort and prognostic factor analysis

**Figure 2:**
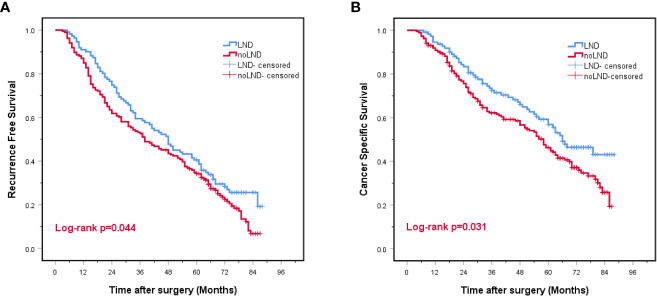
Kaplan-Meier curves for recurrence-free survival **(A)** and cancer-specific survival **(B)** for the entire study cohort. LND, lymph node dissection, Red font means P value < 0.05.

Overall, the LND group had a better prognosis based on the RFS (27.0% vs. 18.3%, p=0.044) and CSS (53.2 vs. 39.8%, p=0.031) than the non-LND group (**Figures 2A, B**).

The univariate analysis revealed that hydronephrosis, urinary cytology, NMIBC, tumor size, tumor Multifocality, LVI, tumor grade, pT stage, pN stage and surgical method were significantly associated with RFS and CSS.

Multivariate analysis revealed that urinary cytology, tumor size, LVI, tumor grade, pT stage, pN stage, surgical method and adjuvant chemotherapy were independent risk factors for RFS. Age, urinary cytology, LVI, tumor grade, pT stage, pN stage, surgical method and adjuvant chemotherapy were independent risk factors for CSS (**Table 2**).

**Table 2:** Univariable and multivariable Cox proportional hazard models predicting recurrence-free survival and cancer-specific survival in the entire study cohort.

Variables	Recurrence-free survival				Cancer-specific survival			
	Univariable analysis		Multivariable analysis		Univariable analysis		Multivariable analysis	
	Unadjusted HR (95% CI)	P value	Adjusted HR (95% CI)	P value	Unadjusted HR (95% CI)	P value	Adjusted HR (95% CI)	P value
Age								
≤66yrs	Reference		Reference		Reference		Reference	
>66yrs	1.278 (0.986-1.655)	0.064	0.851 (0.643-1.126)	0.259	1.092 (0.802-1.487)	0.576	0.715 (0.511-0.999)	**0.049**
Gender								
Male	Reference		Reference		Reference		Reference	
Female	1.084 (0.827-1.420)	0.560	1.053 (0.791-1.400)	0.725	1.102 (0.800-1.518)	0.551	0.967 (0.687-1.362)	0.848
Laterality								
Left	Reference		Reference		Reference		Reference	
Right	1.019 (0.788-1.318)	0.884	0.923 (0.709-1.201)	0.551	0.993 (0.686-1.269)	0.660	0.852 (0.620-1.172)	0.325
Hydronephrosis								
No	Reference		Reference		Reference		Reference	
Yes	2.044 (1.564-2.672)	**<0.01**	1.205 (0.890-1.632)	0.227	2.671 (1.914-3.726)	**<0.01**	1.412 (0.969-2.058)	0.073
Urinary Cytology								
Negative	Reference		Reference		Reference		Reference	
Positive	4.150 (3.132-5.499)	**<0.01**	1.920 (1.383-2.666)	**<0.01**	3.573 (2.709-5.200)	**<0.01**	1.655 (1.130-2.423)	**0.010**
NMIBC								
No	Reference		Reference		Reference		Reference	
Yes	1.944 (1.464-2.583)	**<0.01**	0.987 (0.704-1.383)	0.939	2.248 (1.623-3.113)	**<0.01**	1.193 (0.810-1.760)	0.372
Tumor size								
≤2cm	Reference		Reference		Reference		Reference	
>2cm	3.592 (2.643-4.882)	**<0.01**	1.792 (1.266-2.535)	**0.001**	3.248 (2.269-4.649)	**<0.01**	1.464 (0.971-2.208)	0.069
Tumor Multifocality								
Unifocal	Reference		Reference		Reference		Reference	
Multifocal	2.090 (1.495-2.921)	**<0.01**	1.053 (0.726-1.526)	0.785	2.333 (1.605-3.393)	**<0.01**	1.252 (0.821-1.909)	0.296
LVI								
No	Reference		Reference		Reference		Reference	
Yes	4.724 (3.565-6.259)	**<0.01**	2.708 (1.920-3.820)	**<0.01**	6.221 (4.381-8.833)	**<0.01**	3.030 (1.998-4.594)	**<0.01**
Tumor grade								
Low grade	Reference		Reference		Reference		Reference	
High grade	6.131 (4.391-8.562)	**<0.01**	8.778 (5.668-13.596)	**<0.01**	6.150 (4.087-9.254)	**<0.01**	9.991 (5.855-17.049)	**<0.01**
pT Stage								
pTa/T1/Tis	Reference		Reference		Reference		Reference	
pT2/T3/T4	4.934 (3.413-7.135)	**<0.01**	8.432 (5.324-13.355)	**<0.01**	9.023 (5.164-15.765)	**<0.01**	16.971 (8.812-33.685)	**<0.01**
pN Stage								
pN0	Reference		Reference		Reference		Reference	
pNx	3.957 (2.499-6.265)	**<0.01**	2.279 (1.289-4.027)	**0.005**	5.005 (2.688-9.319)	**<0.01**	2.698 (1.296-5.618)	**0.008**
pN+	9.392 (5.570-15.839)	**<0.01**	5.627 (3.019-8.782)	**<0.01**	10.386 (5.230-20.624)	**<0.01**	5.977 (1.578-9.213)	**<0.01**
Surgical approach								
Laparoscopic RNU	Reference		Reference		Reference		Reference	
Open RNU	1.062 (0.773-1.459)	0.711	1.094 (0.789-1.516)	0.591	0.916 (0.616-1.361)	0.663	0.940 (0.626-1.410)	0.765
Surgical method								
nonLND	Reference		Reference		Reference		Reference	
LND	0.761 (0.581-0.997)	**0.047**	0.285 (0.175-0.463)	**<0.01**	0.699 (0.503-0.972)	**0.033**	0.235 (0.122-0.453)	**<0.01**
Adjuvant chemotherapy								
No	Reference		Reference		Reference		Reference	
Yes	1.036 (0.772-1.389)	0.814	0.542 (0.394-0.744)	**<0.01**	0.990 (0.697-1.405)	0.995	0.505 (0.347-0.736)	**<0.01**
Hospital								
**Yantai**	Reference		Reference		Reference		Reference	
**Tianjin**	1.010 (0.768-1.328)	0.945	0.878 (0.647-1.192)	0.404	0.914 (0.655-1.274)	0.594	0.802 (0.552-1.166)	0.248

HR, hazard ratio; CI, confidence interval; LND, lymph node dissection; NMIBC, non muscle-invasive bladder cancer; LVI, lymphovascular invasion; LN, lymph node; RNU, radical nephroureterectomy; Tis, carcinoma in situ. Black bold means P value < 0.05.

### 3.3 Subgroup analysis according to pT-stage

**Figure 3:**
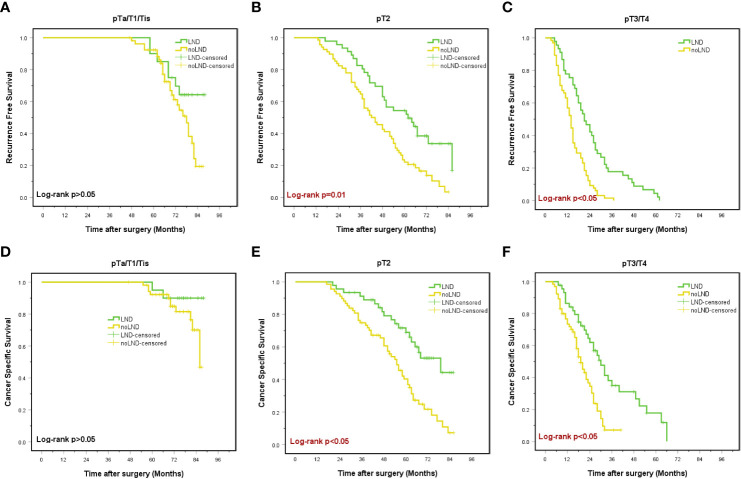
Kaplan-Meier curves for recurrence-free survival **(A-C)** and cancer-specific survival **(D-F)** in subgroups stratified by pathologic tumor (pT) stages. LND, lymph node dissection, Red font means P value < 0.05.

After stratification according to the pT-stage, there were no significant differences in RFS or CSS between the groups in patients with<pT_2_ stage (p>0.05) (**Figures 3A, D**). In contrast, in pT_2-4_ patients, the LND group was associated with overall RFS (18.7% vs. 6.0%, p<0.05), 3-year RFS (50.5% vs. 32.3%, p<0.05) and 5-year RFS(29.7% vs. 12.0%, p<0.05) than the non-LND group. Besides, significant improvement was observed in overall CSS (45.1% vs. 24.1%, p<0.05), 3-year CSS (68.1% vs. 49.6%, p=0.003), and 5-year CSS (51.6% vs. 30.8%, p<0.05) (**Figures 3B, C, E, F**).

Moreover, during univariable and multivariable Cox regression analysis in pT_2-4_ patients, LND was an independent predictor of better RFS (HR 0.255; 95% CI 0.142-0.456; p<0.05) and CSS (HR 0.271; 95% CI 0.137-0.537; p<0.05) than the non-LND group (**Table 3**).

**Table 3:** Univariable and multivariable Cox proportional hazard models predicting recurrence-free survival and cancer-specific survival in patients diagnosed with pT2-4 stage.

Variables	Recurrence-free survival				Cancer-specific survival			
	Univariable analysis		Multivariable analysis		Univariable analysis		Multivariable analysis	
	Unadjusted HR (95% CI)	P value	Adjusted HR (95% CI)	P value	Unadjusted HR (95% CI)	P value	Adjusted HR (95% CI)	P value
Age								
≤66yrs	Reference		Reference		Reference		Reference	
>66yrs	0.927 (0.701-1.227)	0.597	0.897 (0.659-1.221)	0.490	0.791 (0.574-1.091)	0.153	0.734 (0.515-1.046)	0.087
Gender								
Male	Reference		Reference		Reference		Reference	
Female	1.044 (0.777-1.402)	0.777	0.905 (0.663-1.236)	0.531	1.110 (0.794-1.522)	0.541	0.912 (0.638-1.303)	0.611
Laterality								
Left	Reference		Reference		Reference		Reference	
Right	0.970 (0.734-1.282)	0.832	0.868 (0.650-1.159)	0.338	0.961 (0.698-1.324)	0.808	0.842 (0.602-1.178)	0.316
Hydronephrosis								
No	Reference		Reference		Reference		Reference	
Yes	1.916 (1.428-2.570)	**<0.01**	1.208 (0.872-1.674)	0.255	2.241 (1.587-3.164)	**<0.01**	1.292 (0.876-1.905)	0.196
Urinary Cytology								
Negative	Reference		Reference		Reference		Reference	
Positive	3.193 (2.369-4.304)	**<0.01**	1.868 (1.317-2.650)	**<0.01**	2.819 (2.008-3.956)	**<0.01**	1.494 (1.004-2.223)	**0.048**
NMIBC								
No	Reference		Reference		Reference		Reference	
Yes	1.944 (1.464-2.583)	**<0.01**	1.012 (0.705-1.454)	0.946	1.831 (1.308-2.561)	**<0.01**	1.262 (0.840-1.895)	0.263
Tumor size								
≤2cm	Reference		Reference		Reference		Reference	
>2cm	2.613 (1.847-3.698)	**<0.01**	2.036 (1.357-3.054)	**0.001**	2.285 (1.554-3.358)	**<0.01**	1.695 (1.079-2.662)	**0.022**
Tumor Multifocality								
Unifocal	Reference		Reference		Reference		Reference	
Multifocal	2.031 (1.412-2.921)	**<0.01**	1.044 (0.698-1.562)	0.835	2.317 (1.550-3.465)	**<0.01**	1.179 (0.752-1.848)	0.474
LVI								
No	Reference		Reference		Reference		Reference	
Yes	3.072 (2.256-4.184)	**<0.01**	2.985 (2.041-4.365)	**<0.01**	3.629 (2.517-5.230)	**<0.01**	3.329 (2.137-5.188)	**<0.01**
Tumor grade								
Low grade	Reference		Reference		Reference		Reference	
High grade	8.307 (5.374-12.842)	**<0.01**	11.664 (6.935-19.617)	**<0.01**	8.000 (4.887-13.124)	**<0.01**	12.923 (7.119-23.460)	**<0.01**
pN Stage								
pN0	Reference		Reference		Reference		Reference	
pNx	5.916 (3.433-10.195)	**<0.01**	2.077 (1.091-3.954)	**0.026**	6.205 (3.302-11.660)	**<0.01**	2.042 (0.963-4.330)	0.063
pN+	8.310 (4.587-15.053)	**<0.01**	5.117 (2.487-8.139)	**<0.01**	7.702 (3.836-15.466)	**<0.01**	5.368 (2.137-9.012)	-**<0.01**
Surgical approach								
Laparoscopic RNU	Reference		Reference		Reference		Reference	
Open RNU	1.164 (0.827-1.638)	0.384	1.048 (0.740-1.485)	0.792	1.064 (0.709-1.598)	0.763	0.962 (0.635-1.458)	0.856
Surgical method								
nonLND	Reference		Reference		Reference		Reference	
LND	0.589 (0.440-0.788)	**<0.01**	0.255 (0.142-0.456)	**<0.01**	0.521 (0.371-0.733)	**<0.01**	0.271 (0.137-0.537)	**<0.01**
Adjuvant chemotherapy								
No	Reference		Reference		Reference		Reference	
Yes	0.501 (0.370-0.680)	**<0.01**	0.510 (0.366-0.710)	**<0.01**	0.463 (0.323-0.664)	**<0.01**	0.448 (0.303-0.661)	**<0.01**
Hospital								
Yantai	Reference		Reference		Reference		Reference	
Tianjin	1.013 (0.752-1.365)	0.930	0.853 (0.605-1.202)	0.363	0.963 (0.682-1.359)	0.828	0.826 (0.554-1.232)	0.349

HR, hazard ratio; CI, confidence interval; LND, lymph node dissection; NMIBC, non muscle-invasive bladder cancer; LVI, lymphovascular invasion; LN, lymph node; RNU, radical nephroureterectomy; Tis, carcinoma in situ. Black bold means P value < 0.05.

### 3.4 Subgroup analysis stratified by AC

**Figure 4:**
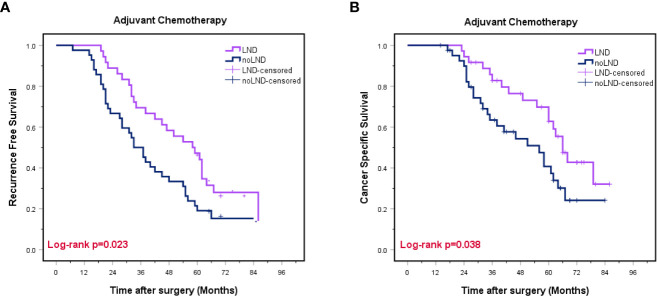
Kaplan-Meier curves for recurrence-free survival **(A)** and cancer-specific survival **(B)** in subgroups stratified by adjuvant chemotherapy. LND, lymph node dissection, Red font means P value < 0.05.

In patients that underwent AC, we found the LND group had a better prognosis in terms of RFS (29.4 vs. 16.7%, p=0.023) and CSS (52.9% vs. 40.5%, p=0.038) than the non-LND group (**Figures 4A, B**).

Univariable and multivariable Cox regression analysis in patients with AC showed that LND was an independent predictor of better RFS (HR 0.172; 95% CI 0.063-0.467; p=0.001) and CSS (HR 0.129; 95% CI 0.033-0.506; p=0.003) compared to the non-LND group (**Table 4**).

**Table 4:** Univariable and multivariable Cox proportional hazard models predicting recurrence-free survival and cancer-specific survival in patients who underwent postoperative adjuvant chemotherapy.

Variables	Recurrence-free survival				Cancer-specific survival			
	Univariable analysis		Multivariable analysis		Univariable analysis		Multivariable analysis	
	Unadjusted HR (95% CI)	P value	Adjusted HR (95% CI)	P value	Unadjusted HR (95% CI)	P value	Adjusted HR (95% CI)	P value
Age								
≤66yrs	Reference		Reference		Reference		Reference	
>66yrs	0.746 (0.451-1.233)	0.253	0.853 (0.466-1.560)	0.605	0.535 (0.292-0.982)	0.043	0.671 (0.311-1.446)	0.309
Gender								
Male	Reference		Reference		Reference		Reference	
Female	1.183 (0.705-1.984)	0.524	1.591 (0.837-3.027)	0.157	1.610 (0.883-22.935)	0.120	1.711 (0.787-3.720)	0.175
Laterality								
Left	Reference		Reference		Reference		Reference	
Right	0.902 (0.545-1.492)	0.688	0.943 (0.511-1.739)	0.851	0.864 (0.474-1.573)	0.632	1.140 (0.522-2.491)	0.743
Hydronephrosis								
No	Reference		Reference		Reference		Reference	
Yes	1.141 (0.690-1.887)	0.606	0.863 (0.459-1.622)	0.647	1.221 (0.670-2.223)	0.514	0.677 (0.298-1.539)	0.352
Urinary Cytology								
Negative	Reference		Reference		Reference		Reference	
Positive	2.796 (1.657-4.720)	**<0.001**	2.122 (1.086-4.147)	**0.028**	2.573 (1.377-4.807)	**0.003**	2.008 (0.925-4.357)	0.078
NMIBC								
No	Reference		Reference		Reference		Reference	
Yes	1.483 (0.864-2.546)	0.153	0.686 (0.334-1.407)	0.303	1.613 (0.858-3.032)	0.138	0.923 (0.384-2.219)	0.858
Tumor size								
≤2cm	Reference		Reference		Reference		Reference	
>2cm	2.279 (1.315-3.951)	**0.003**	1.136 (0.555-2.324)	0.727	1.920 (1.013-3.638)	**0.045**	0.800 (0.341-1.873)	0.607
Tumor Multifocality								
Unifocal	Reference		Reference		Reference		Reference	
Multifocal	1.603 (0.759-3.3831)	0.216	0.825 (0.353-1.932)	0.658	2.094 (0.927-4.732)	0.076	1.017 (0.373-2.770)	0.974
LVI								
No	Reference		Reference		Reference		Reference	
Yes	2.567 (1.532-4.301)	**<0.001**	2.902 (1.410-5.972)	**0.004**	3.244 (1.750-6.016)	**<0.001**	3.027 (1.198-7.651)	**0.019**
Tumor grade								
Low grade	Reference		Reference		Reference		Reference	
High grade	8.576 (3.941-18.660)	**<0.001**	19.271 (7.164-51.840)	**<0.001**	10.484 (3.947-27.853)	**<0.001**	35.749 (9.977-128.089)	**<0.001**
pN Stage								
pN0	Reference		Reference		Reference		Reference	
pNx	4.829 (2.125-10.974)	**<0.001**	2.656 (0.907-7.777)	0.075	4.411 (1.773-10.975)	**<0.001**	3.500 (0.788-15.533)	0.099
pN+	6.909 (2.807-17.006)	**<0.001**	4.337 (1.579-13.891)	**<0.001**	5.747 (2.071-15.948)	**<0.001**	5.891 (2.373-16.328)	**<0.001**
Surgical approach								
Laparoscopic RNU	Reference		Reference		Reference		Reference	
Open RNU	0.909 (0.471-1.754)	0.775	0.585 (0.259-1.321)	0.197	1.226 (0.586-2.568)	0.589	1.112 (0.423-2.925)	0.830
Surgical method								
nonLND	Reference		Reference		Reference		Reference	
LND	0.558 (0.333-0.935)	**0.027**	0.172 (0.063-0.467)	**0.001**	0.530 (0.287-0.979)	**0.042**	0.129 (0.033-0.506)	**0.003**
pT Stage								
pTa/T1/Tis	Reference		Reference		Reference		Reference	
pT2/T3/T4	1.154 (0.281-4.742)	0.842	6.816 (1.050-44.231)	**0.044**	0.791 (0.190-13.287)	0.747	10.487 (1.302-84.502)	**0.027**
Hospital								
Yantai	Reference		Reference		Reference		Reference	
Tianjin	1.259 (0.737-2.151)	0.399	0.716 (0.354-1.450)	0.353	0.410 (0.196-0.857)	**0.018**	0.217 (0.070-0.673)	**0.008**

HR, hazard ratio; CI, confidence interval; LND, lymph node dissection; NMIBC, non muscle-invasive bladder cancer; LVI, lymphovascular invasion; LN, lymph node; RNU, radical nephroureterectomy; Tis, carcinoma *in situ*. Black bold means P value < 0.05.

## 4 Discussion

Due to the rarity of UTUC, most published studies have been limited to small samples with a lack of standardized and prospective randomized trials. The clinical significance of LND during RUN surgery remains subject to debate. During clinical practice, surgeons rarely choose LND due to the burden of surgery (12, 13). According to a study based on the US National Cancer Database, less than 20% of RNUs performed involved LND (14). However, with the development of laparoscopic technology, there is an increasing consensus that LND does not increase the risk of perioperative complications (15, 16). Accordingly, it is necessary to further explore the prognostic significance of LND in UTUC.

In 2017, the European Association of Urology (EAU) guidelines group for non-muscle- invasive bladder cancer published a systematic review, which documented the survival benefit of LND in ≥pT_2_ patients of the renal pelvis. However, the benefits of LAD in ureteral tumors remains uncertain (17). To our knowledge, few studies have hitherto been conducted on UTUC localized to the ureter. It has been established that ureteral UTUC localized to the lower ureter is the most predominant and malignant emphasizing the importance of stratifying patients according to tumor location. Accordingly, the present analysis was innovatively conducted on UTUC cases localized to the lower ureter.

According to the latest European Association of urology guidelines, hydronephrosis and tumor multifocality are high-risk factors for UTUC. Although univariate analysis demonstrated that hydronephrosis and tumor multifocality are the risk factors of prognosis in this study, only pathological T stage and AC were independent risk factors during multivariate analysis. Therefore, we conducted a survival analysis after stratification according to pathological T stage and AC. LND was associated with survival benefits for cases with ≥pT_2_ stage. In addition, during subgroup analysis, the AC cohort showed that the LND group had better survival benefits. Taken together, our findings suggest that AC cannot replace the therapeutic effect of LND in UTUC.

Fan et al. (18) showed that LND could bring survival benefits to patients diagnosed with clinically node-negative UTUC, especially those with T_2-4_ disease. Ikeda et al. (19) also showed that LND improved OS and CSS in pT3 or later stages. However, unlike the present study, they concluded that LND is not beneficial for patients with pT_2_. Besides, Austin et al. (20) showed that extended LND at any clinical stage did not significantly impact OS in cN+ patients with UTUC (20). However, it should be borne in mind that cN+ UTUV is a systemic disease that cannot be controlled by removing the local nodes.

Several limitations found in our study should be acknowledged. Data analyzed in the present study were retrospectively collected from two institutions in China, and the cohort size was relatively small. Further large prospective RCTs are encouraged to substantiate our results. Besides, data on other factors, such as comorbidities (e.g., smoking) and postoperative complications, were not collected. Tobacco is widely acknowledged as an exogenous risk factor for developing UTUC. Indeed, being a smoker at diagnosis increases the risk for disease recurrence and mortality after RNU. The presence of post-operative complications delays AC, increasing the risk for disease recurrence and mortality after RNU. Besides, it has been reported that for tumors of the distal ureter, pelvic dissection could capture only about 75% of lymph node metastases (11). Accordingly, only pelvic lymph node dissection was selected for the present study, which affected the reliability of our findings to a certain extent, emphasizing the need for further studies. Another limitation is the limited sample size. Based on the limited number of samples, it is difficult to conduct subgroup analysis according to the number, size and location of pelvic vessels.

## 5 Conclusion

In conclusion, we provided hitherto undocumented evidence that LND brings survival benefits to patients with UTUC localized to the lower ureter, especially those with ≥pT_2_ stage and AC cohort. In addition, AC cannot replace the therapeutic effect of LND in UTUC.

## Data availability statement

The original contributions presented in the study are included in the article/supplementary material. Further inquiries can be directed to the corresponding author.

## Ethics statement

The authors are accountable for all aspects of the work in ensuring that questions related to the accuracy or integrity of any part of the work are appropriately investigated and resolved. This study was exempt by Institutional Review Board approval.

## Author contributions

YC: study concepts and designs, data acquisition, statistical analysis, and drafted the manuscript. YL: data acquisition, data analysis and interpretation, and manuscript editing. JW: data analysis, manuscript review. CQ: study concepts and designs, data acquisition, data analysis and interpretation, manuscript review. All authors contributed to the article and approved the submitted version.
